# Who is the biological patient? A new gradational and dynamic model for one health medicine

**DOI:** 10.1007/s40656-022-00540-9

**Published:** 2022-11-10

**Authors:** Yael Friedman

**Affiliations:** Centre for Philosophy and the Sciences (CPS), Department of Philosophy, Classics, History of Art and Ideas, University of Oslo, Oslo, Norway

**Keywords:** One health, Organism-environment relation, Patient, Holobiont, Population health, Interdisciplinarity

## Abstract

One Health medicine aims to improve health by focusing on the relations between the health of humans, animals, and the environment. However, One Health does not provide a clear idea of these relations, which are still represented as conceptually separated and not as one health, as the name implies. Inspired by holobiont research, I suggest a new model and conceptual framework for One Health that expands the notion of the biological patient by providing a gradational and dynamic understanding of environments, patients, and their relations. This new model conceptualizes humans and non-humans, individual organisms, and collectives, as belonging to one system that allows for more or less inclusive understandings of patients. As such, it resolves the conceptual tensions of different One Health approaches and supports the implementation of One Health as an interdisciplinary research field.

## Introduction

Illness makes our body apparent to us; suddenly, the pain and discomfort reveal our existence as biological beings. However, does this awareness only reveal our own body, or can it be extended beyond the level of the organism? Western physicians traditionally see the patient as the individual human organism (see, for example: Rosslenbroich, 2016; Sadegh-Zadeh, 2012), whereas everything which is not part of this unit is usually understood simply as ‘environment.’ However, this understanding is critically examined in the medical literature in two ways. First, it is criticised by advocates of population health (PH), public health, and epidemiology, who call on recognizing the population level as irreducible to the individual level (see for example: Giroux, 2021; Institute-of-Medicine­ (US), 2003; Rose, 2001; Rose et al., 2008). Second, it is critiqued by proponents of One Health medicine (OH), which extends the focus of health beyond humans (see for example: Davis & Sharp, 2020; Sironi et al., 2022; Woods et al., 2018a; Zinsstag et al., 2011). OH highlights the relations between the health of humans, animals, and the environment and calls for collaborative research initiatives to improve health both locally and globally. Both PH and OH have become more prominent in light of the current COVID-19 pandemic and the climate crisis (Anderegg et al., 2021; Markovic et al., 2021; Paul et al., 2020; Schmiege et al., 2020; Selbach et al., 2021; Sironi et al., 2022; Viegas, 2021). In a sense, OH can be seen as an extended view of PH’s critical understanding of the group level as a patient, while OH also includes non-humans. I thus see OH as providing a central framework for addressing and developing an extended view of the patient, which constitutes the focus of this article.

A main problem with OH is that it does not present a clear idea of the relation between its components: humans, animals, and the environment. Following Sironi et al. (2022) I distinguish between two main approaches to OH, the prudential and the radical, whose main conceptual differences concern the question of reciprocity in the relations between the three components. Radical OH advocates acknowledge the reciprocal dependence between the three, whereas prudential OH proponents emphasize one-directional relations from animals or from the environment to humans. However, even from the radical point of view, these components are still described as conceptually separated and not as one health, as the name suggests. As such, OH lacks a clear conceptual framework which reflects the interdependence of humans and non-humans. In this article I will provide a suggestion for such a framework by presenting a gradational and dynamic model for OH that accounts for different concepts of patient by implementing new understandings in holobiont research and by rethinking the concept of environment, which so far has received insufficient attention. This model will provide a clear framework which comprises different levels of analysis for patients, environments and their relations. In addition, it provides a theoretical foundation from which to promote OH as an interdisciplinary (instead of a multidisciplinary) research field and therefore has the potential to improve the implementation of OH in medical research and educational programs. The model does not aim to solve ethical questions that OH may bring with it; however, it can be used as a tool for expanding and unifying discussions of OH in different fields.

I open this article by describing the current movement of expanding the concept of the patient led by OH and PH advocates. Then, I show how tensions in central concepts used by the OH movement generate vagueness in the understanding of key relations between humans, animals, and the environment. Inspired by symbionts research, I next develop the features and dynamics of a new patient-environment model, which suggests dynamic part-whole relations between OH components and dissolves their strict boundaries. Finally, I demonstrate how this model opens a novel conceptual space that can help to facilitate the implementation of OH educational programs and research initiatives and improve health.

## Medicine beyond the individual human organism

Traditionally, medicine is organized around the health of the individual human organism. While the discussion often focuses on ‘health’ more generally, it is important to keep in mind that health is typically attributed to a patient. In this article, I discuss and revise the concept of the patient by showing how different ‘levels of patient’ can be identified on the basis of a unified model of dynamic organism-environment relationships.

According to the Cambridge English Dictionary, a “patient is a person who is receiving medical care, or who is cared for by a particular doctor or dentist when necessary” (Patient, 2022). In other words, according to this definition, the patient is an individual human. Etymologically, the word ‘patient’ comes from the Latin ‘patiens,’ from ‘patior,’ to suffer (Neuberger, 1999); that is to say, a patient is an ill person whose suffering compels this person to receive medical help. Modern medicine not only treats those who are already suffering, but also invests in the prevention of possible suffering. Georges Canguilhem famously quoted René Leriche who said that “[H]ealth is life lived in the silence of the organs” (Canguilhem, 2012, p. 91), i.e. where there is no suffering; however, one can ask whether there is suffering that we tend not to hear and mistakenly consider as silence. Alternatively, are there cases in medicine where the indication of suffering is not directly relevant, but where it is still important in accounting for the patient´s health?

Asking who the biological patient is brings to the fore three relevant criticisms of the traditional understanding of this concept. First, one can mention the criticism from an anti-oppressive medical stand. Arriving at medicine from social constructivism and activist groups who defend feminist, anti-racist, and postcolonial approaches, representatives of anti-oppressive medicine argue against the traditional idea of the patient as the white western male body^1^ (see for example: Braidotti, 2013; Mol, 2003; Scott, 1999). Second, some of the advocates of population health (PH) and epidemiology argue for the inclusion of the human group-level (‘public,’ ‘population’ or ‘collective’) as a distinct ‘level of patient’, which is irreducible to individual patients. Finally, One Health (OH) supporters argue for the inclusion of non-humans, such as animals and the environment, as patients (see for example: Davis & Sharp, 2020; FAO et al., 2008; One Health High Level Expert Panel, 2021).

PH and epidemiological studies aim to predict and improve health by incorporating both individual and group level analysis. Since Adolphe Quetelet’s work on the ‘average man’, the group level (or population level) is primarily understood as a statistical entity, and its essence is the intrinsic property of the individuals who comprise it (Krieger, 2012). In addition, when social observations are used, the social determinants of health are understood at the individual level and not at the group level (Giroux, 2021). According to Krieger (Krieger, 2012), this dominant view has been challenged ever since, both empirically and conceptually (see for example: Diez Roux, 2004; Haraway, 2013; Morris, 1955; Rose, 2001; Rose et al., 2008; Sydenstricker, 1933). In short, these critics highlight the central role of the social and political environment and the dynamic and relational nature of populations. Furthermore, they show that group-level characteristics are irreducible to individual characteristics, i.e., the population sum is greater than the parts of its individuals (Giroux, 2021; Krieger, 2012). A salient example of these different patient levels can be found in the case of herd immunity, where immunity, which is gained at the population level, protects non-immune individuals.

Although contributing to different debates, OH can be seen as an extension of this understanding of PH, which provides a more holistic approach that deals with the group level and that includes non-human entities and environments beyond the social environment. This ‘more than human’ dimension of OH is often described as being rooted in an agenda developed by Calvin W. Schwabe (1964, 2004) called One Medicine (OM), which aimed to bring together human and veterinary medicine (Woods et al., 2018b, p. 15). Schwabe first presented the idea of OM in the early 1980s, but it was not until 2002, after a symposium of the Association for Veterinary Epidemiology and Preventive Medicine in honour of his work, that his integrative approach became important (Cassidy, 2018, pp. 193–194). In the same year, the looming threat of zoonoses in the form of a global outbreak of SARS brought wider recognition of the problematic separation of human and animal medicine. A year later, stimulated by the growing concern for the irreversible reality of antibiotic resistance in agriculture, the term ‘One Health’ emerged, which brought into consideration not only animals but also *the environment.* The current COVID-19 pandemic and the global warming crisis have recently brought more attention to OH by demonstrating more clearly than before how we are not only socially linked to each other across the world, but also biologically linked to the world (Diener, 2021).

Traditionally, the individual human organism-environment dichotomy is not explicitly assumed in medicine. Nevertheless, as I have tried to show above, a critical examination of the concept of the biological patient reveals this implicit dichotomy. PH and OH both show that social, biological, and physical environments should not be seen as sharply opposed to humans, and they bring additional crucial perspectives on health and diseases. However, while advocates of PH’s group level argue that relations between the population and individuals are of a part-whole type, in OH the relations between humans, animals, and the environment remain somehow unclear, which can make OH implementation problematic. In the next section, I will identify the gaps which emerge between different OH approaches on this issue.

## One health, multiple understandings

One would expect an initiative titled ‘One Health’ to convey a conceptual unity; instead, as this section will show, OH holds different and inconsistent understandings of the relationship between humans, animals, and the environment. To demonstrate these inconsistencies, I will use observations on the conceptual gaps between OM and early OH, and between early OH to the present projects of OH initiative and OH commission, as they emerge in a chapter about OH’s contemporary history by Angela Cassidy (2018). Following Cassidy, I will analyse the conceptual gaps between the present OH initiative and OH commission and more recent OH projects such as the Joint Quadripartite. I will show how this inconsistency still exists by comparing American OH and international OH, which create two main approaches, the ‘prudential’ and the ‘radical’ approach, as recently presented by Sironi et al. (2022). I agree with the conclusion of these authors that only the development and establishment of the radical OH approach could change our epistemology to include the required sensitivity to animals and the environment, which is central to these approaches. However, I will show that the radical OH approach lacks a consistent model of patients, environments and their relations and still uses diagrams that are not compatible with its epistemology. Therefore, I will point out the need for a new and unitary conceptual framework for OH that will bring these two approaches closer and that will allow for the inclusion of humans, animals, and the environment as part of the same system.

According to Cassidy (2018), the term OM first appeared in the writing of the physiologist Carl F. Schmidt in 1962 in the context of space medicine.^2^ However, it is primarily known for its use in the context of human and animal health, being associated with Schwabe’s work from the 1980s (Woods et al., 2018b). Schwabe’s research on veterinary epidemiology highlighted the importance of veterinary medicine to public health, which later, as described above, influenced the development of OH. The significant difference between OM and the early OH working group’s agenda, conducted by the Wildlife Conservation Society (WCS), lies in the inclusion of the environment by the latter. While OM did not consider the environment as a patient, the WCS asked to shift the focus from medicine to health, which allowed it to bring together OM and the idea of the health of an ecosystem (Cassidy, 2018; Zinsstag et al., 2011). In 2003, the WCS launched the international network of Animal and Human Health for the Environment and Development, AHEAD. They thereby promoted an interconnected network view for the benefit of all species, which they described as ‘One Health’ (Cassidy, 2018).

In many senses, the framework provided by this early OH group was indeed conceptually ahead of later OH initiatives. It is significantly more advanced than the OH Commission and OH Initiative that appeared later. According to Cassidy, these later projects mainly focused on humans’ health and wellbeing, while their attention to animals and the environment remained limited; their idea of zoonosis is mainly understood as one-directional instead of as a reciprocal relation. In other words, the transition is only from animals to humans; by relating to OM and avoiding the contribution of the WCS, the OH Initiative de-emphasized the role of the environment, which in the first place is understood as local to the USA, and is therefore limited (Cassidy, 2018).

In 2008 a new international OH collaboration called the Joint Tripartite (which included The Food and Agriculture Organization of the United Nations, the World Health Organization and the World Organisation for Animal Health), published, together with the World Bank, a “Strategic Framework for Reducing Risk of Infection Diseases at the Animal-Human-Ecosystem Interface” (FAO et al., 2008). This document draws on both the OH commission report and the work of WCS and brought a rise in OH interest outside the USA^3^ (Cassidy, 2018).

In November 2021, the OH High Level Expert Panel (OHHLEP) provided a new definition of OH that was embraced by the Joint Tripartite and the United Nations Environment Programme:

*One Health is an integrated, unifying approach that aims to sustainably balance and optimize the health of people, animals and ecosystems. It recognizes the health of humans, domestic and wild animals, plants, and the wider environment (including ecosystems) are closely linked and inter-dependent. The approach mobilizes multiple sectors, disciplines and communities at varying levels of society to work together to foster well-being and tackle threats to health and ecosystems, while addressing the collective need for clean water, energy and air, safe and nutritious food, taking action on climate change, and contributing to sustainable development. (One Health High Level Expert Panel,* 2021).

This new definition seems to provide a more comprehensive outlook on the relationship between humans, animals, and the environment than its predecessors. As part of this holistic view, in March 2022 the United Nations Environment Programme officially joined the Joint Tripartite, which is now known as the quadripartite (World-Health-Organization, 2022). By working with the OHHLEP’s definition, the quadripartite places a greater focus on non-humans’ health by emphasizing the interdependencies between human and non-humans. OHHLEP also uses a more specific definition of non-humans. Non-humans include, for example, wild animals and plants, which, according to Cassidy (Cassidy, 2018), tend to be neglected compared to house and farm animals. Expanding the view beyond the immediate environment of humans allows for a more inclusive definition of OH. This became more evident when compared to the current OH Commission definition, which emphasizes multi- and transdisciplinarity rather than an integrated approach, that preserves the focus on humans’ health:

*One Health is a collaborative, multisectoral, and trans-disciplinary approach—working at local, regional, national, and global levels—to achieve optimal health (and well-being) outcomes recognizing the interconnections between people, animals, plants and their shared environment.* (One Health Commission 2022).

It is also more holistic compared to the way that the OH initiative describes the concept of OH; the latter emphasizes interdisciplinarity more than the relations between humans, animals, and the environment. It is also evident that the OH’s initiative focus is ultimately on human health:

*The One Health concept is a worldwide strategy for expanding interdisciplinary collaborations and communications in all aspects of health care for humans, animals and the environment. The synergism achieved will advance health care for the 21st century and beyond by accelerating biomedical research discoveries, enhancing public health efficacy, expeditiously expanding the scientific knowledge base, and improving medical education and clinical care. When properly implemented, it will help protect and save untold millions of lives in our present and future generations.* (One Health Initiative 2022).

The gap between the WCS and the Joint Quadripartite, on the one hand, and the OH Commission and the OH initiative, on the other hand, can be described as a conceptual tension. The tension is between the view of OH parts as holistic and interdependent versus the view of OH parts as having a unidirectional relationship, respectively. Recognizing the interconnections between OH parts does not mean reciprocal dependency between them. Sironi et al. (2022) provide a comprehensive analysis of these two accounts which includes not only conceptual but also medical and ethical considerations. According to Sironi et al. (2022, p. 2), the *radical* approach to OH “considers the overall balance of the living eco-system and the environment from a broader perspective than the human one”, while what they call the *prudential* approach “considers prevention and treatment in a broad perspective, but is always, even if indirectly, centered on the human being”.

While proponents of the prudential approach still perceive animals and the environment as having an effect on humans from the outside, proponents of the radical approach see them as part of one system. That is to say, on the prudential approach, there is no difference from the way the patient was seen before OH, whereas on the radical approach, non-humans are included as patients, and this approach therefore provides a new and more extended view on the scope of the biological patient. In their article, Sironi and his colleagues (ibid.) concluded that only the development and establishment of the radical approach could make a difference to non-human health, which both approaches want to improve (as a mean or an aim). Building on their arguments, I would like to suggest that, regardless of their different normative goals, both approaches can benefit from the adoption of a clear conceptual model that extends the understanding of the patient beyond the human patient by seeing humans, animals and the environment as part of one conceptually unified system.

**Fig. 1:**
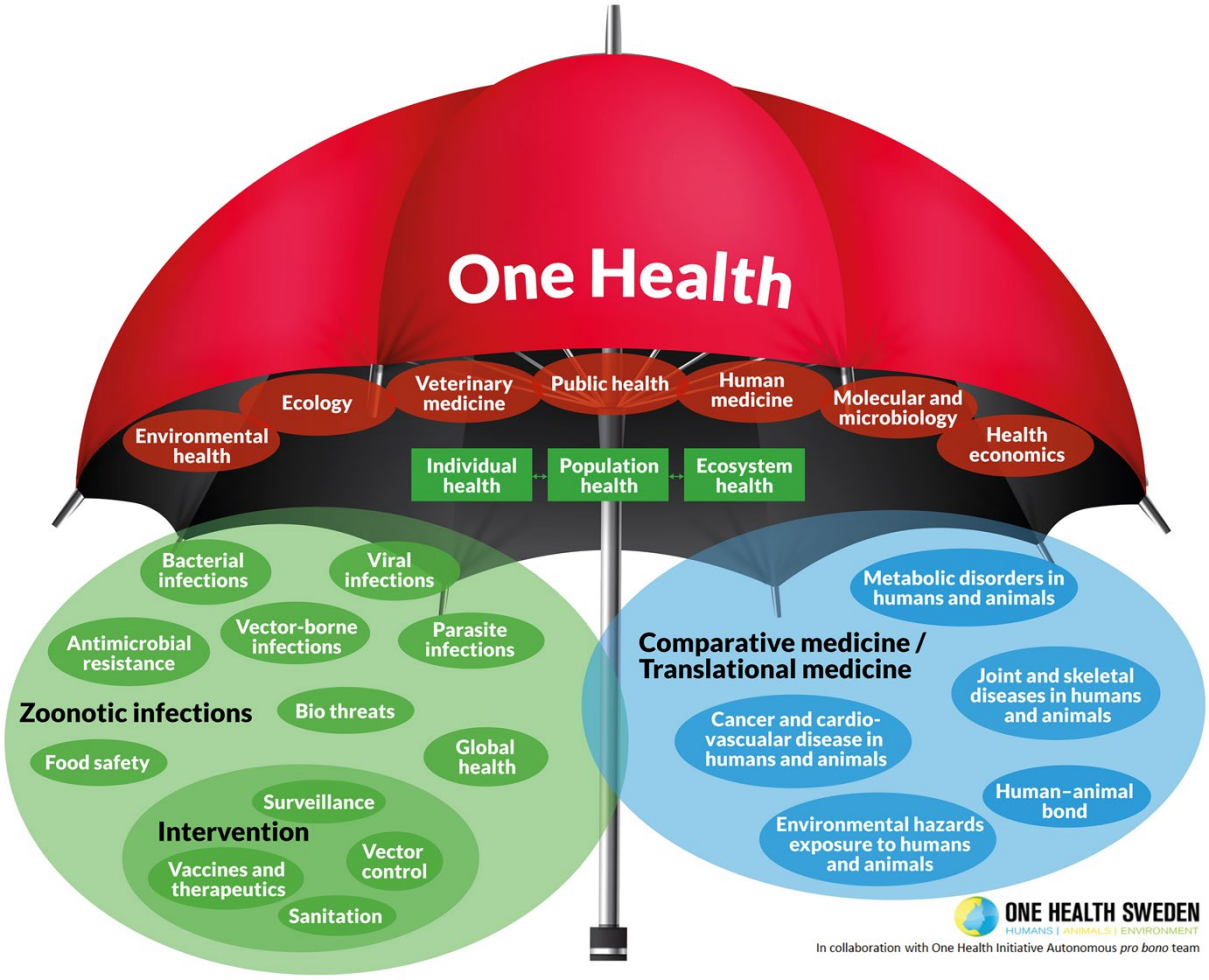
The ‘One Health Umbrella’ developed by the networks ‘One Health Sweden’ and ‘One Health Initiative’ to illustrate the scope of the ‘One Health concept’

**Fig. 2:**
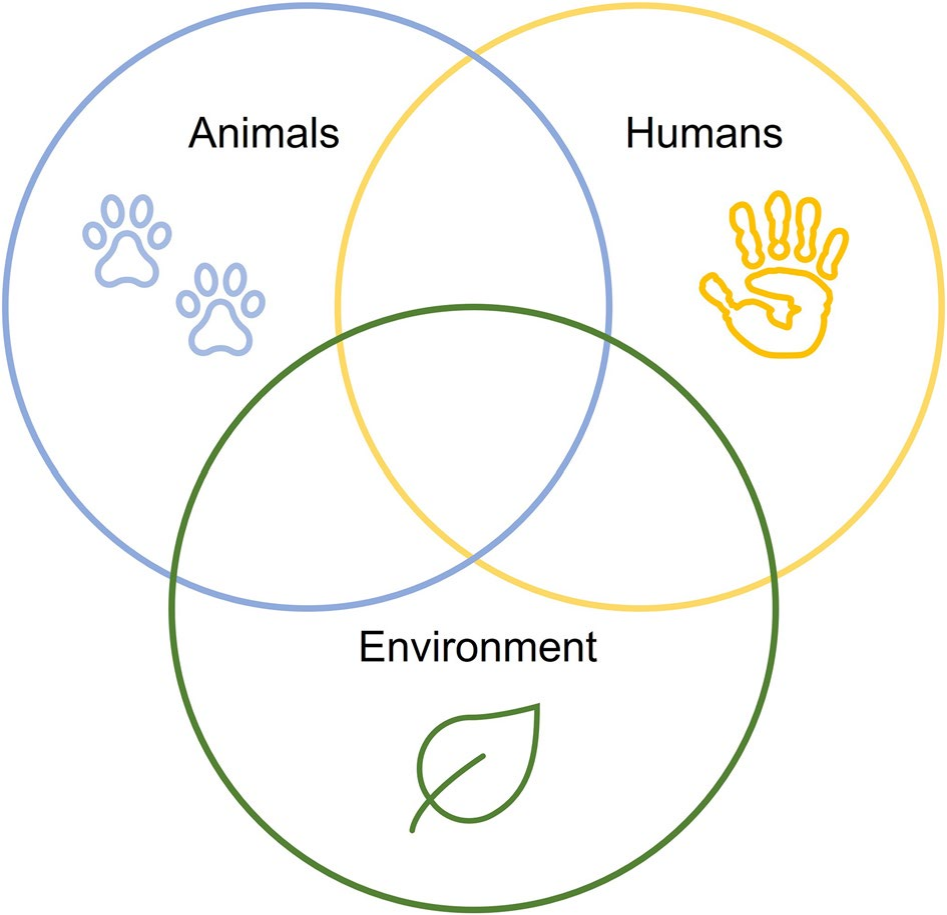
The One Health Medicine triad model of humans, animals, and the environment

When examining the visual representations of OH, it becomes evident that the radical approach to OH lacks a clear model that presents the relations between human and non-humans as one system. Two main diagrams dominate the visuals of OH. First, there is a description of an umbrella made by the Swedish OH initiative (Lerner & Berg, 2015), which covers the different disciplines that collaborate under the OH title (Fig. 1). Second, there is a triad model of humans, animals, and the environment, as represented by three intersected circles (Fig. 2). The triad model is used, with slight graphical variations, in different OH initiatives’ publications and communication channels, such as The One Health European Joint Programme^4^ and the World Health Organization.^5^ The OH umbrella does not account for the relations between the different disciplines and how the interactions between humans, animals, and the environment might be translated into it. The triad model does not explicitly focus on human health, but it shows a conceptual segregation of “bonded, separate and coherent identities” (Davis & Sharp, 2020, p. 2). The triad model depicts animals and the environment as only interfacing with humans. This interface is the space in which threats to human health emerge, and so the triad model only represents health threats anthropocentrically (ibid.). Thus, neither of these two visuals captures the integrative conceptual dimension required by the radical OH approach (a dimension that would also benefit the prudent OH approach).

**Fig. 3:**
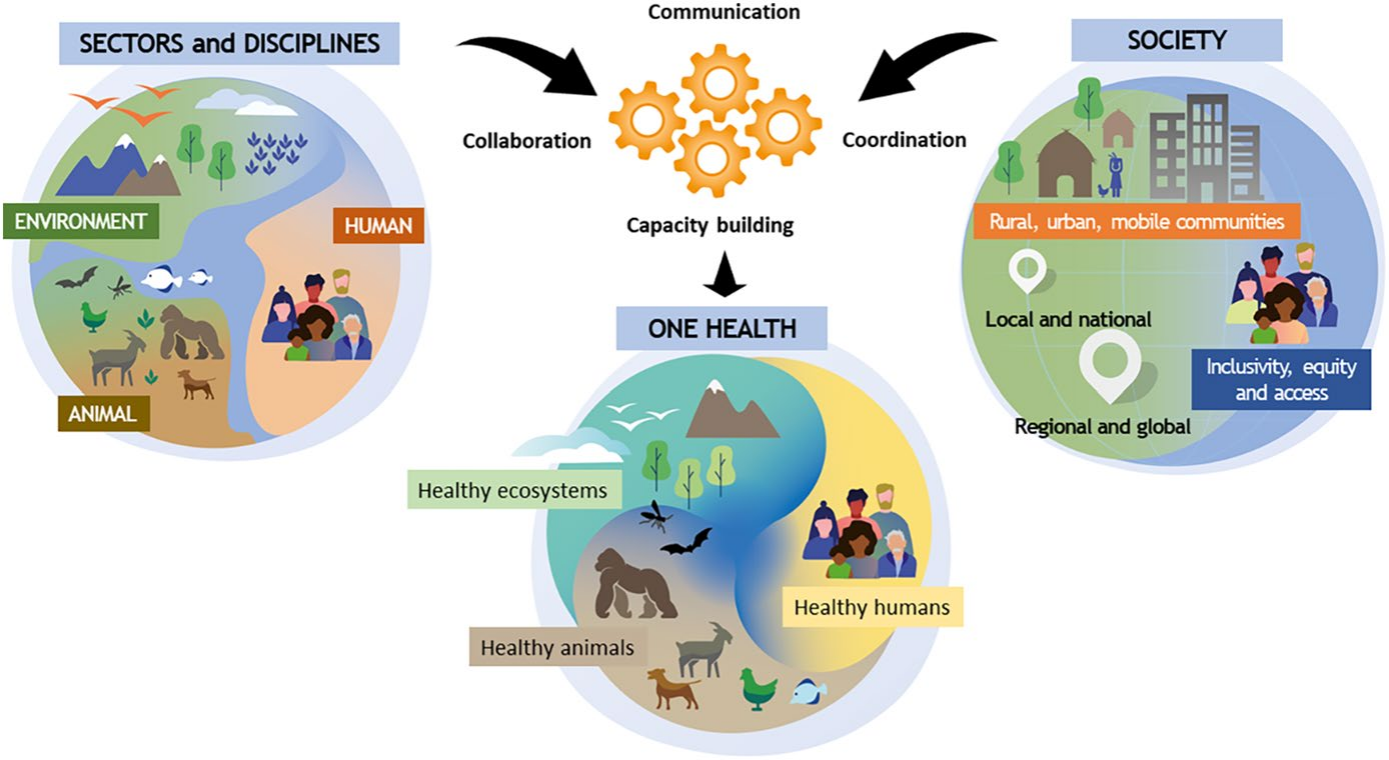
The One Health Medicine diagram developed by One Health High-Level Expert Panel (OHHLEP)

A new diagram (Fig. 3) accompanies the OHHLEP’s novel definition which regards human health, animal health, and environmental health as three—somehow interacting—thirds of the same whole (One Health High Level Expert Panel, 2021). While this is a step in the right direction (also in comparison to the sharp separation shown between the sectors and disciplines on the left circle), it still presents a clear separation between the three. By preserving this divided (trinary) mode of thought, the OHHLEP also fails to align with the unitary vision of OH. This could be an obstacle to the future development of OH educational programs that bring together different research fields and include all the mentioned components. OH education has been criticized as being mainly focusing on human health and veterinary medicine, while neglecting environmental studies (Essack, 2018; Ogunseitan, 2022; Villanueva‐Cabezas et al., 2020). I hold that OH needs a new unitary model of the relations between humans, animals, and the environment, that allows both the relevant components and the relations between them to be understood in a more integrated way.

I will therefore suggest a new and unified model that can be utilized by both the prudential and the radical approaches of OH and that allows for shifts in focus between humans and non-humans. A model that permits such shifts would also provide a suitable framework for a more nuanced discussion of the ethics of OH, e.g. when the anthropocentrism of dominant approaches is questioned (see, on the current discussion of OH ethics: Johnson & Degeling, 2019, 2020; Lederman & Capps, 2020; Lederman et al., 2021).

The suggested OH model aims to bridge the chasm between the individual human organism, animals, and the environment, while focusing on the concept of the patient. One way to formulate this chasm is in terms of the organism/environment dichotomy^6^: on the one hand, we have the human organism, and, on the other hand, everything else, i.e., the environment, which includes all other species (as well as the social environment). How does one go beyond the organism/environment dichotomy to provide a unitary conceptual system for OH? The next section explains how holobiont research can inspire such a model.

## Holobionts as a conceptual aid for a unitary model of OH

A possible way to go about conceptualising a new holistic model of OH is to draw inspiration from biological discussions in which organism-environment relations have already been challenged. From the early twentieth century, biological research has been extensively devoted to this issue. Some research was directly focused on the question of boundaries, such as the works of Lancelot Law Whyte (Whyte, 1949), John Scott Haldane (Haldane, 1917), Joseph Needham (Needham, 1936), and Marcel Prenant (Prenant, 1938).^7^Discourse on the essence of biological individuality and how it relates to the organism also plays a crucial role in the consideration of this dichotomy (see for example: Child, 1915; Chiu & Eberl, 2016; Clarke, 2013; Haber & Odenbaugh, 2009; Landecker, 2017; Skillings, 2016). Different types of collectives have also been considered as units of living beings, which affect environment boundaries. Concepts such as holocoen (*Holozön*) and biocoenosis (*Biozönose)*^8^(Jax, 1998, 2020)*,* ecosystem (Tansley, 1935), and Gaia (Lovelock, 1979; Margulis & Sagan, 1997) are only a few examples of such inclusive living beings. Here, I focus on a popular contemporary concept: the holobiont, understood as the host together with its symbiotic microbes (Margulis & Fester, 1991; Rosenberg & Zilber-Rosenberg, 2013). In the following I will show how holobiont research can contribute a conceptual ground for developing a more holistic model of OH.

Since researchers and practitioners of medicine are both already invested in the implementation of holobiont research in their practice, holobionts bear the potential to bridge biological and medical understandings of patients, organisms, animals, and environments. Understood as a holobiont, the human host and its symbiotic microbes can maintain a stable homeostasis, which is described as the “healthy state” of the holobiont (Van De Guchte et al., 2018). Some multifactorial pathologies are now also associated with imbalanced microbiota. Obesity (Rosenberg & Zilber-Rosenberg, 2019), inflammatory bowel diseases (Durack & Lynch, 2019), type 2 diabetes (Liu & Lou, 2020), depression (Yang et al., 2020), and anorexia nervosa (Ghenciulescu et al., 2021) are just a few examples of pathologies associated with different compositions of gut microbiota (these are often understood as unhealthy and called “dysbiosis”). One medical intervention which has been tested as a treatment and prevention of these conditions is fecal microbiota transplantation (FMT), a procedure in which gut microbiota from healthy donors are transplanted into individuals who have the condition or are in a risk group. When medical practitioners treat patients with FMT, they see them as both organisms and holobionts, and they track the changes in the microbiota of the holobionts to treat the symptoms of the human organisms. Furthermore, the human holobiont is described in different ways depending on *medical context*; for example, gynecologists and gastrologists will be interested in different microbiota associated with the same human host.

Holobiont research can be seen as a reconsideration of our boundaries and a problematization of the biological picture of individuality and collectivity. In a paper that describes the contribution of the Rosenberges to the development of the idea of holobionts as individual consortia, the philosopher Ehud Lamm suggests that the holobiont perspective brought at least three foundational arguments (Lamm, 2018, p. 12): “Symbiosis is developmentally plastic, the primary unit of evolution transcends the individual organism, and Lamarckian inheritance, via the inheritance of the adapted microbiome, is alive and kicking”. Thus, holobiont research provides both *temporality* and *scientific context* as crucial components for defining the boundaries of a living being. This, in turn, opens up new understandings of the notions of collectivity (Gissis et al., 2017) and environment.

Holobiont research puts a spotlight on the *temporality* of the living being’s composition. This idea echoes Canguilhem’s understanding in his famous essay on the living [being] and its milieu; as he writes, “[t]he biological relationship between the being and its milieu is a functional relationship, and thereby a mobile one” (Canguilhem, 2009, p. 111). There are at least two kinds of changes that can occur over time in a symbiotic relationship. The first is when a ‘microbiota turns over’, meaning that the identity of the microbial partners changes throughout the life of the host (Suárez & Stencel, 2020). The living being then partially changes over time through an exchange process with microbes that were previously external to it. The second possible change in a symbiotic relationship is when microbes turn from being beneficial to being pathogenic (or vice versa) (Lamm, 2018). These changes can also be gradual or quantitative. Take, for example, the dynamic nature of the microbe population in the human gut, which are part of a human’s holobiont. Imbalance in the number of *eubacterium Hudsonia* (which is related to the improvement of diabetes mellitus) or the lack of *F. prausnitzii* (which improves insulin sensitivity) can affect the development of type 2 diabetes (Durack & Lynch, 2019; Liu & Lou, 2020). Another example is *Escherichia coli*, a significant member of the gut microbiota, which in some cases may cause life-threatening infections, while probiotic strains of the same bacterium may prevent the recurrence of bladder infections in women (Pitlik & Koren, 2017). Thus, the nature of symbiotic relations can ‘turn over’, changing what we may consider as the living being across time. Both these types of changes mark temporality as a crucial factor in a dynamic model of organism-environment relations. In other words, the composition of patients might look different over time.

According to the philosopher Thomas Pradeu, ‘organism’ and ‘biological individual’ are not necessarily synonyms but may refer to different things depending on scientific context (Pradeu, 2016a, 2016b). Pradeu argues that one should distinguish between physiological and evolutionary individuality. This distinction maps more specific arguments regarding the unit of reference and what it includes. For example, regarding physiological individuality, some scientists focus on the unit of immunity (Pradeu, 2016b, 2020; Tauber, 2017) while others emphasize the unit of metabolism (Dupré & O'Malley, 2009). Consequently, the notion of individuality and its boundaries can be affected by the scientific context which is at play. Similarly, in the context of OH the notion of the patient and its boundaries might be different when moving from one scientific perspective to another.

The ontological status of the holobiont has significance for the perspective from which one defines the relations between the entities in question. Holobionts are likely to include both coevolved and non-coevolved groupings, as well as both physiologically dependent and independent species (Lloyd & Wade, 2019). *That is to say, symbiotic relations are not necessarily symmetrical and can be regarded differently depending on whether they are seen from the perspective of the host or the symbionts*. The new ‘microbe-relative’ perspective (Suárez & Stencel, 2020) adds to the traditional host-centric definition of holobionts. When OH prioritizes the human patient, it can still benefit from the microbe-relative perspective, for example when the cure of the host depends on the cure of some dysfunctional microbial population. By the same token, we can benefit from other non-human-relative perspectives, as in the case of zoonoses. An OH model should allow for a shift in focus from human to non-human without alteration to the conceptual system.

Holobiont research brings with it possibilities for new understandings of the relations between the patient and the environment, the human and the non-human. Both in holobiont and OH views, what is considered as the patient may not be immediately clear and intuitive. First, the same body includes different dimensions of a patient that may require different treatments. Second, since the boundaries are dynamic, and what is considered environment can become part of the patient, we can think about different levels of patients beyond the boundaries of the human organism. Similar to the holobiont patient that changes according to the medical context, OH requires a dynamic understanding of the patient that will provide some common ground for different research fields to partake. In addition, temporality should also be taken into account in an OH model, as the interactions of human and non-humans, individuals and groups have mutual effects on each other that can change their boundaries and compositions. Thus, a new OH model should take into consideration both the *variation* in scientific perspective and *temporality* when defining the patient.

Further, since parts of the environment can sometimes be seen as part of the patient, the model should allow for a gradational understanding of the environment, namely as different exteriors that correspond to different dimensions of the patient. In OH, humans, animals, and the environment can be understood as constituents of dynamic part-whole relations with changing (and gradual) boundaries depending on the patient in focus and the context of discourse. The patient (human or non-human) should not be seen as sharply opposed to their exterior but rather as on a spectrum with it: they are distinguished from some of the exteriors, as seen from a specific point in time and medical context, while they are interlocked with other exteriors that may constitute the patient when seen from other relevant perspectives.

## The model

In the following, I introduce the proposed conceptual model for OH which comprises four levels of patients. To create a spectrum of environments, I use the general term ‘exterior’. The model aims to serve as a unitary tool that conceptualizes and compares different notions and relations of patients, which includes humans, animals, and the environment from a variety of scientific and temporal standpoints.

### Domains

**Fig. 4:**
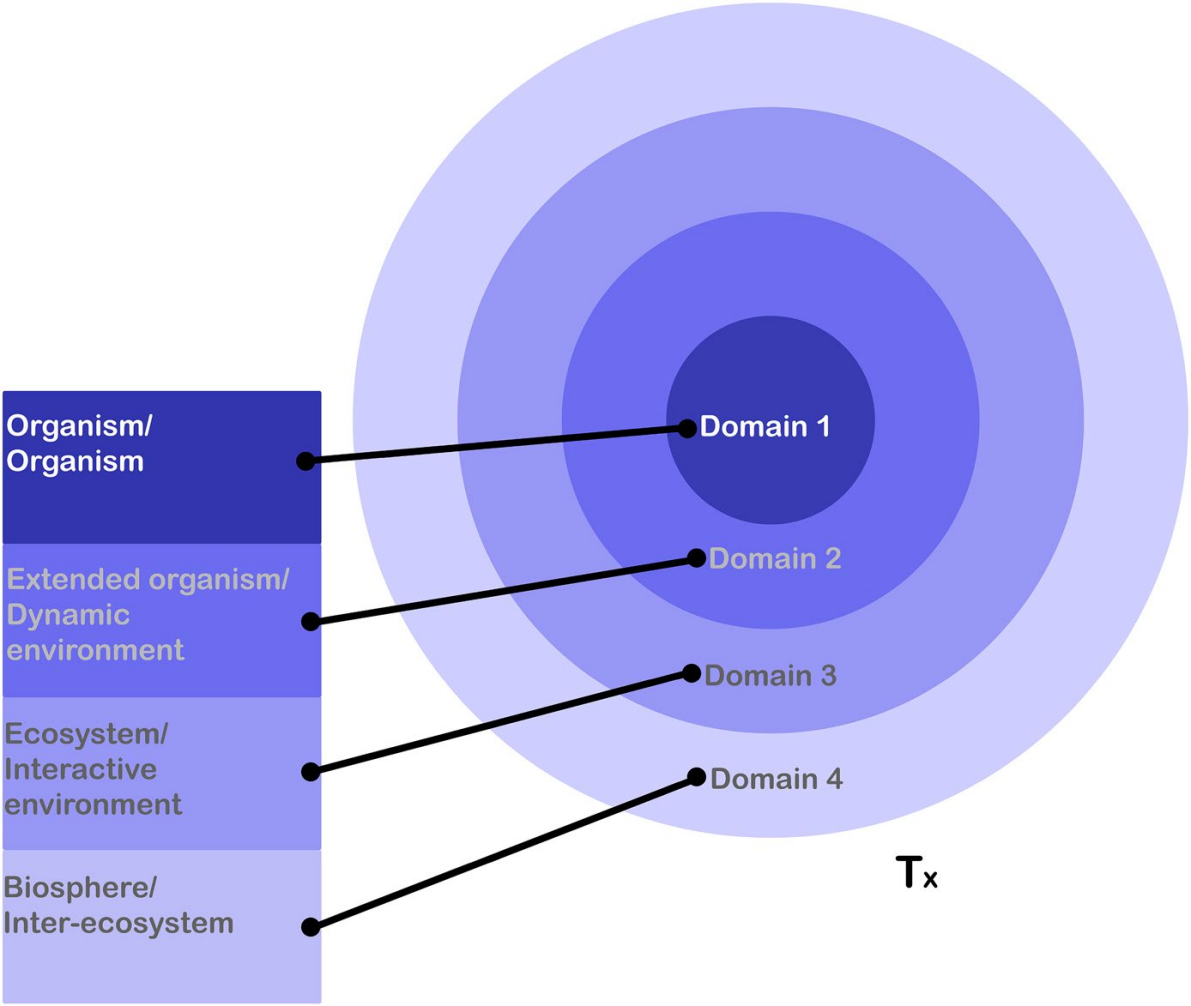
The patient and its exterior model. The four concentric spheres represent the four domains of the model. Each domain contains a patient level and an exterior level. The model presents the state of affairs in a specific time (T_X_). Domain 1: An organism (patient level) can be unicellular or multicellular, prokaryote or eukaryote, and without symbionts. An organism (exterior level) is exterior to the organism’s systems, organs, and cells. So, e.g., a human being can be seen as an organism patient level and as exterior to its organs. In the same way, a wild boar can be seen as an organism patient level and as exterior to its organs. Domain 2: An extended organism is an organism with “extended phenotypes” (biotic or abiotic), which I call the dynamic environment. Take, for example, a human and its microbes, or a wild boar and its microbes as extended organism patient level; in both examples the microbes are the dynamic environment. Domain 3: An ecosystem includes different kinds of human and non-human groups and their interactive environment. The interactive environment includes the conditions and circumstances with which a patient directly interacts that are understood as external. Here the patient level could be, for example, a human population living in a city C where a population of wild boars is also living. The interactive environment in this case is city C, including the wild boar population. Continuing the example of the wild boar, the patient is a population of wild boars living in a human populated city C, and the interactive environment is city C including the human population. Domain 4: The biosphere is the ecosystem of all ecosystems. The inter-ecosystem includes all the other ecosystems outside the one in question, with which it interacts. According to the example, the ecosystem is city C, including the human and the wild boar populations, and the inter-ecosystem is all the ecosystems outside of this city with which it interacts directly and indirectly, such as the region, the country, the continent, and the planet in which it partakes. Together they constitute the biosphere which is the patient level

The model consists of four domains, which build up a spectrum of relations between the patient and its exterior. The domains are represented as concentric spheres in Fig. 4. The peripheral spheres include the central spheres, so the relations between the center and the periphery are exclusion relations, while the relations between the periphery and the center are inclusion relations. In other words, the domains are nested within one another.

Each domain includes a patient level and an exterior level. Domain 1 includes the organism patient and the organism exterior. Domain 2 contains the extended organism patient and the dynamic environment exterior. Domain 3 incorporates the ecosystem patient and the interactive environment exterior. Domain 4 is the sum of all domains and constitutes the biosphere patient and the inter-ecosystem exterior.

The patient in the different domains not only has exteriors but also consists of exteriors. The model is organised so that a patient of a domain (x) and an exterior of a following domain (x + 1) constitute the boundaries of the patient in the following domain (x + 1). According to this rule, the organism (domain 1) and the dynamic environment (domain 2) together constitute the extended organism (domain 2); the extended organism (domain 2) and the interactive environment (domain 3) define an ecosystem (domain 3); and the ecosystem (domain 3) and the inter-eco-system (domain 4) form the biosphere (domain 4). That is to say, the patient both interacts with and is made of what is outside of the organism (‘the exterior’), depending on the patient in focus. When the patient in focus is the extended organism, we can say that the extended organism is constituted by the organism and the dynamic environment and engages with the interactive environment. Thus, the relation between the patient and the organism ‘exterior’ can be seen as both binary and non-binary. The model presents the state of affairs in time *t*.

### Patients

I suggest distinguishing between four different levels of patient, namely the organism, the extended organism, the ecosystem, and the biosphere. In the following I will address each of them and explain how patients may be understood in all of these levels in the different domains (which I will call ‘spheres’, referring to Fig. 4 above).

As we saw previously, the discourse on holobionts made it possible to perceive a biological individual not only as an organism but also as a collective of a host and its symbionts, opening the patient boundaries to some level of the exterior. As discussed above, individuality can be understood and identified both above and below the organism level. It is a concept that can cross the model’s domains. Elements that find themselves below the organism level may sometimes be understood as individuals, e.g., cells. In domain 2, I borrow J. Scott Turner’s (2000) term ‘extended organism’, which can also be understood, in some cases, as individuality above the organism level. I use the term ‘extended organism’ and not ‘holobiont’, as I leave room also for the abiotic extended organism, which is not included within the concept of holobiont (see more in the next section). Unlike Turner’s extended organism that does not account for different exteriors, the extended organism here refers more specifically to the extension of the organism to the dynamic environment.

An organism can be unicellular or multicellular, prokaryote or eukaryote, and without symbionts.^9^ An extended organism includes the organism and its extended phenotypes, such as microbes. The degree of overlap between the organism and the extended organism is dependent on the scientific perspective and temporality. Whenever the organism and the extended organism refer to the same entity, they will be represented as completely overlapping spheres. That can be the case when there are no extended phenotypes, in which case the dynamic environment is “empty”. Take, for example, a germ-free mouse (which has no other biotic or abiotic extended phenotypes); the germ-free mouse extended organism is, in this case, equal to the mouse organism.

The ecosystem patient level includes different kinds of human and non-human groups and their interactive environment. One can define different kinds of groups and sub-groups according to various criteria; some are only relevant for humans (e.g., gender or ethnicity), some only for non-humans (e.g., belonging to a specific herd or pack), and others are relevant for both (e.g., population based on geography, age, sex, kinship).^10^ Second, as previously discussed regarding holobionts, the relations between the host and the symbionts in a holobiont are not always symmetrical (from the viewpoint of a microbe, the holobiont can be either an extended organism or an ecosystem). This OH model allows not only for microbe-relative perspectives but also for non-anthropocentric perspectives more generally. That is to say, the same ecosystem can be understood from the perspectives of different organisms.^11^The ecosystem includes the interactive environment which consists of aspects such as material conditions and culture (social behaviours in animals).

Last comes the biosphere level, which allows for a holistic view on life. Although not all entities share an environment (see below on the definition of environment), they may affect one another indirectly. As stressed by Lovelock’s Gaia theory (Lovelock, 1979), we should be able to discuss the biosphere as an integral whole. As the ecosystem of all ecosystems, the biosphere includes the inter-ecosystem exterior.

The model is organised so that the patient can be a collective and can contain non-living entities. The decision to form the model in this way reflects the idea that the boundaries of patients and exteriors are flexible, not necessarily “sterile”, and above all are context dependent. From the most exclusive to the most inclusive, all patients have different exteriors. Extending the boundaries of the patient beyond the organism also requires a detailed understanding of its exterior, as will be developed in the following subsection.

### Exteriors

In this section, I suggest distinguishing between four different exteriors, namely the organism, the dynamic environment, the interactive environment, and the inter-ecosystem. In doing so, I will discuss the new terminology of exteriors and explain how the exteriors correspond to the different spheres of the model.

The history of biology is rich in examples of different uses of the term ‘environment’ and different terms that express the concept of environment, such as milieu and circumstances. The concept of environment created a unification of various terminology (Pearce, 2010). The organism is the focal point of the concept of environment, which may allow us to think of environment as a graded concept which possesses different degrees of inclusiveness and exclusiveness. We may, for example, conceptually narrow down the idea of environment from the physical, biological, and social world in general to the environment of the specific organism. The model presents the idea of environment as a spectrum. However, I believe that using the term ‘environment’ to describe both the spectrum in general and a section of it can cause confusion. Therefore, in the following, l use the term 'exterior' to discuss what is left outside of the patient addressed. I take ‘exterior’ to be a general term that includes the environment which is specific to an organism/extended organism as well as the physical, biological and social conditions that are not specific to it. It is important to note that I use the term ‘exterior’ relative to the patient in focus.

The term ‘organism’ has a dual function in the model, and it is defined both as patient and as exterior. The organism functions as exterior to its parts, for example in the form of internal systems (such as the nervous system), organs or cells. In other words, the organism exterior is what in the literature is often called ‘internal environment’. Since the model offers different ‘internal environments’ that correspond to different patients, I prefer to avoid this term. I will keep using the term ‘environment’ in relation to both organisms and extended organisms, but I differentiate between the organism’s environment and the extended organism’s environment, as specified in the following.

Inspired by symbionts research, I suggest distinguishing between two different relations which occur in the traditional organism-environment dyad: interactive relations and dynamic relations. Dynamic relations are relations between the organism (domain 1) and the exterior that may become part of the (extended) organism (domain 2). Interactive relations are those between the extended organism (domain 2) and the exterior that is part of the ecosystem (domain 3). The dynamic relations reflect the nature of the extended organism as enduring, i.e. as an entity that has different temporal parts throughout its life-span (see: Triviño & Suárez, 2020, p. 15). The extended organism is built up from a host organism and acquires entities that are exterior to it and undergo change over time. Entities that can be acquired in this sense form the exterior of the organism, or what I call the dynamic environment. While the extended organism consists of the organism and the dynamic environment, the extended organism interacts with a different exterior, which I call the interactive environment. Like the traditional concept of environment, the interactive environment includes the conditions and the circumstances with which an extended organism interacts, and it is understood as external, including physical, biological, and social domains.

It is important to note that interactive relations exist between an extended organism and other entities (biotic or abiotic), while the dynamic relation forms the extended organism itself. The dynamic environment thus relates to the organism while the interactive environment relates to the extended organism. These two different relations are only distinguishable when we allow biological individuality to be understood as a collective, as is the case for the extended organism. In current literature, this collective notion of individuality is often employed (see for example: Gilbert et al., 2012; Gissis et al., 2017; Suárez & Stencel, 2020), and thus the distinction between the dynamic and the interactive environment becomes of great use and significance.^12^

The dynamic environment contains factors that may be considered as part of the extended organism, depending on the scientific and social context. For the host organism the symbionts can be seen as “extended phenotypes”, while for the symbionts the host is a source of niche (Gilbert, 2017). For example, in a nosological sense, a diseased microbiota is an observable characteristic of an extended organism (the host). Accordingly, the microbes are organic phenotypes of the host organism. I use the notion of the ‘extended phenotype’ differently from the way that it was introduced by the evolutionary biologist Richard Dawkins (1982), who was interested in the effect of the gene outside of one’s body but did not regard organisms as phenotypes of each other. Similarly, the extended phenotype can also be inorganic, as in the case of cyborgs (Haraway, 2006), if the inorganic extension is significant to the extended organism health.^13^ Inorganic phenotypes can be, for example, pacemakers and stents.

The exterior of the fourth domain is an inter-ecosystem exterior. Outside the ecosystem in question, there are other ecosystems that include biological, social, and physical conditions. The inter-ecosystem exterior includes all the other ecosystems outside the one in question, with which it interacts. All the ecosystems together constitute the biosphere.

## The model’s uses and benefits

The model provides a unified conceptual ground for OH in which patients and the environment constitute each other as one system with different levels of parts and wholes that invite different levels of analysis. The model extends the notion of patient beyond the individual human organism, and it includes both animals and the environment as well as different collectives as relevant levels of analysis in the same system, which are epistemologically equal and non-reducible to one another. In other words, it does not prioritize some patient levels over other patient levels, and it allows the necessary level (or levels) to be addressed on a case-by-case basis. In this sense, it is a unitary model that provides a pluralistic view, rather than a reductionistic view as often characterizes unitary models.

The model is conceptually different from the way that OH is currently presented—both on the prudential and the radical approach—where non-humans constitute a threat to humans from the “outside”. The model opens up a novel conceptual space for OH in the understanding of disease development by directing more attention to the dynamic relations and dependencies of different patient levels and thereby allowing a focus on possibly neglected causal relations, which can lead to new interventions or affect the treatment path.

To give an example, one can think of how the suggested model can highlight new possible ways of understanding the development, treatment, and prevention of COVID-19, through reconsideration of the biological patient. First, OH brings to light the connection between the development of COVID-19 (and other infectious and respiratory diseases) in relation to environmental factors (see for example: Asif et al., 2022; Wang et al., 2022; Weaver et al., 2022), which according to the suggested model are understood as the interactive environment. Here, following the prudential approach, the interactive environment is considered external to the patient. However, using the model, one can also think about the interactive environment from the radical perspective, i.e., as part of a (different) patient level when considering the health of an ecosystem and the biosphere during the pandemic. Research shows the temporary reduction of greenhouse gases and other toxic emissions due to the preventive measures of COVID-19, like lockdowns and travel restrictions (see for example: Guevara et al., 2021, 2022), which can positively affect the health of ecosystems and biospheres (even if only temporally). Giving more attention to the ecosystem and the biospheres can lead to the differentiation between health rates in various levels of analysis, from which one can also draw conclusions for general health management that balances health at different levels. This is not to suggest taking any of the specific measures of lockdowns and travel restrictions to protect the ecosystem and the biosphere, but to promote informed decisions with different patient levels in mind. This view changes the focus from the disease threat to the dynamic maintenance of health, which is the aim of OH. It also allows professionals from different fields to collaborate for the sake of the health of the ecosystem or the biosphere, and not only for the health of humans.

Second is an example of possible new prevention paths for COVID-19, which does not focus on humans. Immunity for COVID-19 is mainly discussed in relation to the vaccination of human organisms and the herd immunity effect in the human population, while a non-human interactive environment is understood as carrying a risk for infection. New research on cross-species vaccination (Warimwe et al., 2021) opens the theoretical possibility of cross-species immunity, where the interactive environment is seen as potentially protective. Discussing ecosystem immunity requires a different, unified, level of analysis since, theoretically, an ecosystem’s immunity is not reducible to a specific population’s immunity (herd immunity) or an organism’s or an extended organism’s immunity. The model allows us to conceptualize the interactive environment as both internal and external to the patient, taking into consideration the different patient levels. Thus, the model supports the aim of both OH approaches to promote health. It also promotes a PH agenda by taking into consideration the population level in a non-reductionistic way.

Lastly, the suggested model could also help to highlight new possible treatment paths that are not focused on the organism and so far, have gotten little attention. According to the World Health Organization (WHO), COVID-19 symptoms in humans commonly include fever, dry cough, or fatigue (World-Health-Organization, 2020). A severe case of the disease might include symptoms such as shortness of breath, persistent pain, or pressure in the chest (ibid.). These symptoms appear in and are experienced by the human organism. Research on other non-human organisms, like horses, dogs and pigs, shows they experience different symptoms of COVID-19 (Korath et al., 2022). Thus, attributing symptoms to a patient relies on the common understanding of the patient as an organism. However, some research on COVID-19, which so far has remained marginal, looks into a possible link between the development of the disease and the gut microbiota (Albrich et al., 2022; Giannos & Prokopidis, 2022; Sarkar et al., 2021). This possibility might contribute to the explanation of why patients with conditions associated with dysbiosis (such as type 2 diabetes, cardiovascular disorders, and the elderly) are more vulnerable and develop more severe cases of COVID-19. If this is the case, the treatment and prevention is not only directed to the host organism but should also take into account the symbiont community. According to Giannos and Prokopidis (2022), clinical trials are investigating whether supplementation of anti-inflammatory bacterial species and/or fecal transplantation would accelerate gut microbiota restoration and long COVID-19 rehabilitation. That is to say, possible treatments aim to cure the symbiont community (the dynamic environment), highlighting the extended organism at the patient level rather than the human organism level as it is usually understood. The model also allows for shifting the perspective from humans to microbes in its center while still keeping microbe-human interaction as a relevant patient level.

The example of COVID-19 shows that while some levels of analysis are being considered, others get much less attention or are not understood as an integral part of the patient but as external to it, which matches the prudential approach to OH. While all levels of analysis would not always be relevant for treatment or prevention regarding a specific disease, the model allows them to be equally and systematically considered, which makes room for the radical OH approach. By doing so, one not only conceptualizes who the patient is but also how the environment is understood as part of the patient. It allows us to simultaneously see both disease and health at different levels and expand the care for the patient beyond the narrow biomedical scope.

Furthermore, the model can facilitate the promotion of OH as having an interdisciplinary, rather than a multidisciplinary, research agenda. One of the barriers to the implementation of OH is the difficulty of bringing together educational programs from different relevant fields. OH is mainly discussed in medicine and veterinary education, where there is a tendency to neglect the environmental component (Essack, 2018; Ogunseitan, 2022; Villanueva‐Cabezas et al., 2020). Using the model, it will be harder to neglect any of the three components, as they entangle and constitute each other. For example, when discussing zoonotic diseases through the model, one would have to take into consideration domain 3, in which the interaction between extended organisms (human and non-human) takes place. Here, one cannot separate the material condition in which the interaction happens. That is to say, environmental factors are entangled in the patient level, rather than being external to it.

Moving from a model of different systems with some overlapping interfaces to a more unified model can promote greater synergy between different kinds of expertise and allow new pathways of information flow to promote health at different patient levels. Translation of knowledge and terminology is always a complicated task, but it is necessary for OH development. The model’s terminology of different patient levels and exteriority is a first step for creating a common ground for further translations between the disciplines. Instead of focusing on the difference between disciplines, it brings to the fore the discussion on levels of patients, opening up in a more systematic way for future research medical inference beyond the traditional level of the individual organism and beyond the anthropocentric focus of PH.

As Ogunseitan (2022) highlights, one way to deal with the gap between different areas of knowledge is to merge disciplines into an independent department. As a conceptual basis for OH, the model may contribute to breaking down traditional disciplinary boundaries and seeing OH as a unified field of studies. At the same time, the model brings the two OH approaches, the prudential and the radical, closer together; both can use the model regardless of their ethical stance. Minimizing the conceptual conflicts within OH also lends support to its implementation.

As such, the model does not provide a direction for solving ethical questions in this area. The organism in the center of the model could be human or non-human, and the level of patient on which one focuses is not implied by the model. However, it can serve as a comparative tool to assess the distribution of health between different patient levels. Using the model, one can track how the attention and resources of OH initiatives are distributed. It can be used to research inequalities both between humans and non-humans and between different humans’ groups and populations, for example the inequalities in health that emerge between ecosystems that differ in their wealth. For the radical approach to OH, the model can thus provide the conceptual framework needed to facilitate the development of a new ethical system, as this approach aims for.

## Concluding remarks

Who is the biological patient? In this article, I have shown that OH can benefit from being conceptualized as one system that allows for the inclusion of humans, animals, and the environment in a gradational and dynamic way and that accounts for different patient levels. Inspired by holobiont research, I have suggested that the environment can be seen as part of the patient (human and non-human) and not simply external to it. Extending the concept of the patient to include animals and the environment as well as collectives can facilitate a new understanding of health and disease and form a unified conceptual ground that narrows, and potentially closes, the gap between the prudential and radical OH approaches. More conceptual clarity for OH supports the end goal of improving health.

## Data Availability

Not applicable.

## Footnotes

1 This anti-oppressive criticism is also directed against OH. See, for example, the criticisms directed against the OH universalism of western health values (Craddock & Hinchliffe, 2015; Friese & Nuyts, 2017; Rock 2017) and the way it disregards the social, cultural, and political context of health (Wallace et al., 2015; Woldehanna & Zimicki, 2015). As one can see, this literature is strongly related to post-humanistic criticism on medicine, which can be viewed as a contemporary evolution of anti-oppressive medicine. Like the radical approach to OH's (presented below), posthumanism calls for going beyond the anthropocentric focus and include the health of cyborgs, animals, and ecosystems. While OH and posthumanism can interlock, they are rooted in different traditions and have different rationales, requiring separate discussions. Here I will only focus on OH.

2 Space medicine is the practice of medicine in outer space, including the health maintenance of space travellers.

3 OH existed outside of the USA already, for example in the Swiss Tropical and Public Health Institution (Zinsstag et.al., 2005).

4 The One Health European Joint Programme diagram https://onehealthejp.eu/about/.

5 The World Health Organization diagram: https://twitter.com/who/status/959023059737939968.

6 The idea of conceptualizing humans, animals, and the environment as one system may appear similar to the Developmental System Approach (DST) and its attempt to think beyond dichotomies (Ingold,^2003^; Oyama, 2006; Oyama, 2001). DST's goal is to do biology without privileged causes, and therefore, it aims to go beyond the nature/nurture dichotomy. So, in DST literature, 'environment' is another word for nurture. However, the suggested model focuses on the living being/environment dichotomy to discuss different patient levels.

7 An extensive account of this issue has been given by Alejandro Fábregas Tejeda at a talk titled ‘Drawn, erased, re‐negotiated: The organism‐environment boundary in early twentieth‐century biology’, July 13th 2021, ISHPSSB Biennial Meeting, Cold Spring Harbor Laboratory, United States [online meeting].

8 According to Jax (Jax, 1998, 2020), ‘biocoenosis’ refers to a living community, a unit of life which exists at a higher level of organization than the organism. ‘Holocoen’ refers to the biocoenosis and its environment, the biotope.

9 The model does not account for a specific view on organisms and allows us to see organisms from different perspectives, e.g., physiological, metabolic, or immunological.

10 According to the suggested model, the population level is not an intermediate level between the extended organism and the ecosystem, as the interactive environment is already included in the population level. Public and population health are therefore considered to be on the ecosystem level. Although using different terminology, PH is included in the suggested model of OH.

11 When the model is centered around a microbe organism, the dynamic environment could be, for example, only the host's immune system, and the interactive environment could include the whole host organism. In this regard, the model allows us to include sub-organismal units.

12 In cases in which the organism is not extended, the model will show the dynamic environment as an empty set ∅, and the organism would interact with the interactive environment directly.

13 What is considered significant depends on the context and should have a clear threshold.

## References

[CR1] Albrich WC, Ghosh TS, Ahearn-Ford S, Mikaeloff F, Lunjani N, Forde B, Suh N, Kleger G-R, Pietsch U, Frischknecht M, Garzoni C, Forlenza R, Horgan M, Sadlier C, Negro TR, Pugin J, Wozniak H, Cerny A, Neogi U, O’Toole PW, O’Mahony L (2022). A high-risk gut microbiota configuration associates with fatal hyperinflammatory immune and metabolic responses to SARS-CoV-2. Gut Microbes.

[CR2] Anderegg, W. R., Abatzoglou, J. T., Anderegg, L. D., Bielory, L., Kinney, P. L., & Ziska, L. (2021). Anthropogenic climate change is worsening North American pollen seasons. *Proceedings of the National Academy of Sciences*. Advance online publication. 10.1073/pnas.2013284118 10.1073/pnas.2013284118 PMC7896283 33558232

[CR3] Asif Z, Chen Z, Stranges S, Zhao X, Sadiq R, Olea-Popelka F, Peng C, Haghighat F, Yu T (2022). Dynamics of SARS-CoV-2 spreading under the influence of environmental factors and strategies to tackle the pandemic: A systematic review. Sustainable Cities and Society.

[CR4] Braidotti R (2013). The posthuman.

[CR5] Canguilhem, G. (2009). The living and its milieu. In: *Knowledge of life* (pp. 98–120). Fordham University Press.

[CR6] Canguilhem, G. (2012). *On the normal and the pathological* (Vol. 3). Springer.

[CR7] Cassidy, A. (2018). Humans, other animals and ‘one health’in the early twenty-first century. In: Abigail Woods, Michael Bresalier, Angela Cassidy & Rachel Mason Dentinger (Eds.), *Animals and the shaping of modern medicine* (pp. 193–245). Springer. 10.1007/978-3-319-64337-3

[CR8] Child CM (1915). Individuality in organisms. University of Chicago Press.

[CR9] Chiu L, Eberl G (2016). Microorganisms as scaffolds of host individuality: An eco-immunity account of the holobiont. Biology & Philosophy.

[CR10] Clarke E (2013). The multiple realizability of biological individuals. The Journal of Philosophy.

[CR11] Craddock S, Hinchliffe S (2015). One world, one health? Social science engagements with the one health agenda. Social Science and Medicine.

[CR12] Davis A, Sharp J (2020). Rethinking one health: Emergent human, animal and environmental assemblages. Social Science & Medicine.

[CR13] Dawkins R (1982). The extended phenotype.

[CR14] Diener, L. (2021). COVID-19 und seine Umwelt: Von einer Geschichte der Humanmedizin zu einer ökologischen Medizingeschichte? *NTM Zeitschrift für Geschichte der Wissenschaften, Technik und Medizin,**29*(2), 203–211. 10.1007/s00048-021-00299-3 10.1007/s00048-021-00299-3 PMC8054680 33871662

[CR15] Diez Roux AV (2004). The study of group-level factors in epidemiology: Rethinking variables, study designs, and analytical approaches. Epidemiologic Reviews.

[CR16] Dupré, J., & O’Malley, M. A. (2009). Varieties of living things: Life at the intersection of lineage and metabolism. *Philosophy, Theory, and Practice in Biology*. Advance online publication. 10.3998/ptb.6959004.0001.003

[CR17] Durack J, Lynch SV (2019). The gut microbiome: Relationships with disease and opportunities for therapy. Journal of Experimental Medicine.

[CR18] Essack SY (2018). Environment: The neglected component of the one health triad. The Lancet Planetary Health.

[CR19] FAO, OIE, WHO, Coordination, U. S. I., UNICEF, & BANK, W. (2008). *Contributing to one world, one health*. https://www.fao.org/3/aj137e/aj137e00.htm.

[CR20] Friese C, Nuyts N (2017). Posthumanist critique and human health: How nonhumans (could) figure in public health research. Critical Public Health.

[CR21] Ghenciulescu, A., Park, R. J., & Burnet, P. W. J. (2021). The gut microbiome in anorexia nervosa: Friend or foe? *Frontiers in Psychiatry*. Advance online publication. 10.3389/fpsyt.2020.611677 10.3389/fpsyt.2020.611677 PMC7835121 33510660

[CR22] Giannos P, Prokopidis K (2022). Gut dysbiosis and long COVID-19: Feeling gutted. Journal of Medical Virology.

[CR23] Gilbert SF, Gissis SB, Lamm E, Shavit A (2017). Rethinking parts and wholes. Landscapes of collectivity in the life sciences.

[CR24] Gilbert SF, Sapp J, Tauber AI (2012). A symbiotic view of life: We have never been individuals. The Quarterly Review of Biology.

[CR25] Giroux É (2021). Can populations be healthy? Perspectives from Georges Canguilhem and Geoffrey Rose. History and Philosophy of the Life Sciences.

[CR26] Gissis S, Lamm E, Shavit A (2017). Landscapes of collectivity in the life sciences.

[CR27] Guevara M, Jorba O, Soret A, Petetin H, Bowdalo D, Serradell K, Tena C, Denier van der Gon H, Kuenen J, Peuch V-H (2021). Time-resolved emission reductions for atmospheric chemistry modelling in Europe during the COVID-19 lockdowns. Atmospheric Chemistry and Physics.

[CR28] Guevara M, Petetin H, Jorba O, Denier van der Gon H, Kuenen J, Super I, Jalkanen JP, Majamäki E, Johansson L, Peuch VH, Pérez García-Pando C (2022). European primary emissions of criteria pollutants and greenhouse gases in 2020 modulated by the COVID-19 pandemic disruptions. Earth System Science Data.

[CR29] Haber M, Odenbaugh J (2009). The edges and boundaries of biological objects. Biological Theory.

[CR30] Haldane JS (1917). Organism and environment as illustrated by the physiology of breathing.

[CR31] Haraway, D. (2006). A cyborg manifesto: science, technology, and socialist-feminism in the late twentieth century (1985). In Joel Weiss, Jason Nolan, Jeremy Hunsinger & Peter Trifonas (Eds.), *The international handbook of virtual learning environments* (pp. 117–158 ). Springer. 10.1007/978-1-4020-3803-7_4.

[CR32] Haraway D (2013). When species meet.

[CR33] Ingold, T. (2003). From complementarity to obviation: On dissolving the boundaries between social and biological anthropology, archaeology and psychology. In S. Oyama, R. D. Gray, & P. E. Griffiths (Eds.), *Cycles of contingency: Developmental systems and evolution. *MIT Press.

[CR34] Institute-of-Medicine ­(US). (2003). *Understanding population health and its determinants* (978-0-309-08704-9). The Future of the Public Health in the 21st Century, Issue. https://www.ncbi.nlm.nih.gov/books/NBK221225/?report=classic.

[CR35] Jax K (1998). Holocoen and ecosystem—on the origin and historical consequences of two concepts. Journal of the History of Biology.

[CR36] Jax K (2020). “Organismic” positions in early German-speaking ecology and its (almost) forgotten dissidents. History and Philosophy of the Life Sciences.

[CR37] Johnson J, Degeling C (2019). Does one health require a novel ethical framework?. Journal of Medical Ethics.

[CR38] Johnson J, Degeling C (2020). More philosophical work needed in one health on ethical frameworks and theory. Journal of Medical Ethics.

[CR39] Korath ADJ, Janda J, Untersmayr E, Sokolowska M, Feleszko W, Agache I, Adel Seida A, Hartmann K, Jensen-Jarolim E, Pali-Schöll I (2022). One health: EAACI position paper on coronaviruses at the human-animal interface, with a specific focus on comparative and zoonotic aspects of SARS-CoV-2. Allergy.

[CR40] Krieger N (2012). Who and what is a “population”? Historical debates, current controversies, and implications for understanding “population health” and rectifying health inequities. The Milbank Quarterly.

[CR41] Lamm, E. (2018). Big dreams for small creatures: Ilana and Eugene Rosenberg’s path to the hologenome theory. In O. Harman & M. R. Dietrich (Eds.), *Dreamers, visionaries, and revolutionaries in the life sciences* (pp. 288–304). University of Chicago Press. 10.7208/chicago/9780226570075.001.0001

[CR42] Landecker H, Lidgard S, Nyhart LK (2017). Metabolism, autonomy, and individuality. Biological individuality.

[CR43] Lederman Z, Capps B (2020). One health ethics: A response to pragmatism. Journal of Medical Ethics.

[CR44] Lederman Z, Magalhães-Sant’Ana M, Voo TC (2021). Stamping out animal culling: From anthropocentrism to one health ethics. Journal of Agricultural and Environmental Ethics.

[CR45] Lerner H, Berg C (2015). The concept of health in one health and some practical implications for research and education: What is one health?. Infection Ecology & Epidemiology.

[CR46] Liu, Y., & Lou, X. (2020). Type 2 diabetes mellitus-related environmental factors and the gut microbiota: Emerging evidence and challenges. *Clinics*. Advance online publication. 10.6061/clinics/2020/e1277 10.6061/clinics/2020/e1277 PMC6945290 31939557

[CR47] Lloyd EA, Wade MJ (2019). Criteria for holobionts from community genetics. Biological Theory.

[CR48] Lovelock J (1979). Gaia: A new look at life on earth.

[CR49] Margulis L, Fester R (1991). Symbiosis as a source of evolutionary innovation: Speciation and morphogenesis.

[CR50] Margulis L, Sagan D (1997). Slanted truths: Essays on Gaia, symbiosis and evolution. Springer-Verlag.

[CR51] Markovic S, Rodic A, Salom I, Milicevic O, Djordjevic M, Djordjevic M (2021). COVID-19 severity determinants inferred through ecological and epidemiological modeling. One Health.

[CR52] Mol, A. (2003). *The body multiple*. Duke University Press. 10.1215/9780822384151.

[CR53] Morris JN (1955). Uses of epidemiology. British Medical Journal.

[CR54] Needham J (1936). Order and life.

[CR55] Neuberger J (1999). Do we need a new word for patients? Lets do away with "patients". BMJ (clinical Research Ed.).

[CR56] Ogunseitan OA (2022). One health and the environment: from conceptual framework to implementation science. Environment: Science and Policy for Sustainable Development.

[CR57] One Health High Level Expert Panel. (2021). Retrieved 14 April 2022 from https://www.who.int/news/item/01-12-2021-tripartite-and-unep-support-ohhlep-s-definition-of-one-health.

[CR58] One Health Commission. Retrieved 14 April 2022 from https://www.onehealthcommission.org/en/why_one_health/what_is_one_health/.

[CR59] One Health Initiative. Retrieved 15 April 2022 from https://onehealthinitiative.com/about/.

[CR60] Oyama, S., Griffiths, P. E., & Gray, R. D. (2001). Introduction: What is developmental systems theory. In: *Cycles of contingency: Developmental systems and evolution* (pp. 1–11).

[CR61] Oyama S, Neumann-Held EM, Rehmann-Sutter C (2006). Boundaries and (constructive) interaction. Genes in development: Re-reading the molecular paradigm.

[CR62] Patient. In. (2022). *dictionary.cambridge.org*. Retrieved 13.4.2022, from https://dictionary.cambridge.org/dictionary/english/patient.

[CR63] Paul E, Brown GW, Ridde V (2020). COVID-19: Time for paradigm shift in the nexus between local, national and global health. BMJ Global Health.

[CR64] Pearce T (2010). From ‘circumstances’ to ‘environment’: Herbert Spencer and the origins of the idea of organism–environment interaction. Studies in History and Philosophy of Science Part C: Studies in History and Philosophy of Biological and Biomedical Sciences.

[CR65] Pitlik SD, Koren O (2017). How holobionts get sick—toward a unifying scheme of disease. Microbiome.

[CR66] Pradeu T (2016). The many faces of biological individuality. Biology & Philosophy.

[CR67] Pradeu T (2016). Organisms or biological individuals? Combining physiological and evolutionary individuality. Biology & Philosophy.

[CR68] Pradeu, T. (2020). Philosophy of immunology. *Cambridge University Press*. Advance online publication. 10.1017/9781108616706

[CR69] Prenant M (1938). Biology and marxism.

[CR70] Rock MJ (2017). Who or what is ‘the public’ in critical public health? Reflections on posthumanism and anthropological engagements with one health. Critical Public Health.

[CR71] Rose G (2001). Sick individuals and sick populations. International Journal of Epidemiology.

[CR72] Rose GA, Khaw K-T, Marmot M (2008). Rose's strategy of preventive medicine: The complete original text.

[CR73] Rosenberg, E., & Zilber-Rosenberg, I. (2013).* The hologenome concept: Human, animal and plant microbiota*. Springer. 10.1007/978-3-319-04241-1

[CR74] Rosenberg, E., & Zilber-Rosenberg, I. (2019). The hologenome concept of evolution: medical implications. *Rambam Maimonides Medical Journal*. Advance online publication. 10.5041/RMMJ.10359 10.5041/RMMJ.10359 PMC6363370 30720424

[CR75] Rosslenbroich B (2016). The significance of an enhanced concept of the organism for medicine. Evidence-Based Complementary and Alternative Medicine.

[CR76] Sadegh-Zadeh, K. (2012). The bio-psycho-social agent. In: *Handbook of analytic philosophy of medicine, *2nd ed., Vol. 1, (pp. 124–165). Springer. 10.1007/978-94-017-9579-1.

[CR77] Sarkar A, Harty S, Moeller AH, Klein SL, Erdman SE, Friston KJ, Carmody RN (2021). The gut microbiome as a biomarker of differential susceptibility to SARS-CoV-2. Trends in Molecular Medicine.

[CR78] Schmiege D, Perez Arredondo AM, Ntajal J, Minetto Gellert Paris J, Savi MK, Patel K, Yasobant S, Falkenberg T (2020). One Health in the context of coronavirus outbreaks: A systematic literature review. One Health.

[CR79] Schwabe CW (1964). Veterinary medicine and human health.

[CR80] Schwabe CW (2004). Keynote address: The calculus of disease-importance of an integrating mindset. Preventive Veterinary Medicine.

[CR81] Scott AL (1999). Paradoxes of holism: Some problems in developing an antioppressive medical practice. Health.

[CR82] Selbach C, Vanhove MPM, Mouritsen KN (2021). The evolutionary ecology of SARS-CoV-2: A missing perspective in the One Health approach. Transboundary and Emerging Diseases.

[CR83] Sironi VA, Inglese S, Lavazza A (2022). The “One Health” approach in the face of Covid-19: How radical should it be?. Philosophy, Ethics, and Humanities in Medicine.

[CR84] Skillings D (2016). Holobionts and the ecology of organisms: Multi-species communities or integrated individuals?. Biology & Philosophy.

[CR85] Suárez, J., & Stencel, A. (2020). A part-dependent account of biological individuality: Why holobionts are individuals and ecosystems simultaneously. *Biological Reviews*. Advance online publication. 10.1111/brv.12610 10.1111/brv.12610 32406121

[CR86] Sydenstricker E (1933). Health and environment.

[CR87] Tansley AG (1935). The use and abuse of vegetational concepts and terms. Ecology.

[CR88] Tauber AI, GIssis SB, Lamm E, Shavit A (2017). Immunity, a collective phenomenon. Landscapes of collectivity in the life sciences.

[CR89] Triviño V, Suárez J (2020). Holobionts: Ecological communities, hybrids, or biological individuals? A metaphysical perspective on multispecies systems. Studies in History and Philosophy of Science Part C: Studies in History and Philosophy of Biological and Biomedical Sciences.

[CR90] Turner JS (2000). The extended organism : The physiology of animal-built structures.

[CR91] Van De Guchte, M., Blottière, H. M., & Doré, J. (2018). Humans as holobionts: Implications for prevention and therapy. *Microbiome*. Advance online publication. 10.1186/s40168-018-0466-8 10.1186/s40168-018-0466-8 PMC5928587 29716650

[CR92] Viegas, S. (2021). Climate change and the need of a one health approach—from science to policy. *European Journal of Public Health*. Advance online publication. 10.1093/eurpub/ckab164.271

[CR93] Villanueva-Cabezas JP, Rajkhowa A, Campbell AJ (2020). One Health needs a vision beyond zoonoses. Transboundary and Emerging Diseases.

[CR94] Wallace RG, Bergmann L, Kock R, Gilbert M, Hogerwerf L, Wallace R, Holmberg M (2015). The dawn of structural one health: A new science tracking disease emergence along circuits of capital. Social Science & Medicine.

[CR95] Wang D, Wu X, Li C, Han J, Yin J (2022). The impact of geo-environmental factors on global COVID-19 transmission: A review of evidence and methodology. Science of the Total Environment.

[CR96] Warimwe GM, Francis MJ, Bowden TA, Thumbi SM, Charleston B (2021). Using cross-species vaccination approaches to counter emerging infectious diseases. Nature Reviews Immunology.

[CR97] Weaver AK, Head JR, Gould CF, Carlton EJ, Remais JV (2022). Environmental factors influencing COVID-19 incidence and severity. Annual Review of Public Health.

[CR98] Whyte LL (1949). The unitary principle in physics and biology.

[CR99] Woldehanna S, Zimicki S (2015). An expanded one health model: Integrating social science and one health to inform study of the human-animal interface. Social Science & Medicine.

[CR107] Woods, A., Bresalier, M., Cassidy, A., & Mason Dentinger, R. (2018a). *Animals and the shaping of modern medicine: One health and its histories*. Springer. 10.1007/978-3-319-64337-3. 29437323

[CR100] Woods, A., Bresalier, M., Cassidy, A., & Mason Dentinger, R. (2018b). Introduction: Centring animals within medical history. In: *Animals and the shaping of modern medicine: One health and its histories* (pp. 1–26). Springer. 10.1007/978-3-319-64337-3_1.

[CR102] World-Health-Organization. (2020). *Coronavirus disease (COVID-19)*. https://www.who.int/news-room/questions-and-answers/item/coronavirus-disease-covid-19.

[CR103] World-Health-Organization. (2022). *UN Environment Programme joins alliance to implement One Health approach*. Retrieved 14 April 2022 from https://www.who.int/news/item/18-03-2022-un-environment-programme-joins-alliance-to-implement-one-health-approach.

[CR104] Yang Z, Li J, Gui X, Shi X, Bao Z, Han H, Li MD (2020). Updated review of research on the gut microbiota and their relation to depression in animals and human beings. Molecular Psychiatry.

[CR105] Zinsstag J, Schelling E, Waltner-Toews D, Tanner M (2011). From “one medicine” to “one health” and systemic approaches to health and well-being. Preventive Veterinary Medicine.

[CR106] Zinsstag J, Schelling E, Wyss K, Mahamat MB (2005). Potential of cooperation between human and animal health to strengthen health systems. Lancet.

